# Gene-Expression Signature Predicts Postoperative Recurrence in Stage I Non-Small Cell Lung Cancer Patients

**DOI:** 10.1371/journal.pone.0030880

**Published:** 2012-01-23

**Authors:** Yan Lu, Liang Wang, Pengyuan Liu, Ping Yang, Ming You

**Affiliations:** 1 Department of Physiology and the Cancer Center, Medical College of Wisconsin, Milwaukee, Wisconsin, United States of America; 2 Departments of Laboratory Medicine and Pathology, Mayo Clinic, Rochester, Minnesota, United States of America; 3 Departments of Health Sciences Research, Mayo Clinic, Rochester, Minnesota, United States of America; 4 Department of Pharmacology and Toxicology and the Cancer Center, Medical College of Wisconsin, Milwaukee, Wisconsin, United States of America; Queen Elizabeth Hospital, Hong Kong

## Abstract

About 30% stage I non-small cell lung cancer (NSCLC) patients undergoing resection will recur. Robust prognostic markers are required to better manage therapy options. The purpose of this study is to develop and validate a novel gene-expression signature that can predict tumor recurrence of stage I NSCLC patients. Cox proportional hazards regression analysis was performed to identify recurrence-related genes and a partial Cox regression model was used to generate a gene signature of recurrence in the training dataset −142 stage I lung adenocarcinomas without adjunctive therapy from the Director's Challenge Consortium. Four independent validation datasets, including GSE5843, GSE8894, and two other datasets provided by Mayo Clinic and Washington University, were used to assess the prediction accuracy by calculating the correlation between risk score estimated from gene expression and real recurrence-free survival time and AUC of time-dependent ROC analysis. Pathway-based survival analyses were also performed. 104 probesets correlated with recurrence in the training dataset. They are enriched in cell adhesion, apoptosis and regulation of cell proliferation. A 51-gene expression signature was identified to distinguish patients likely to develop tumor recurrence (Dxy = −0.83, P<1e-16) and this signature was validated in four independent datasets with AUC >85%. Multiple pathways including leukocyte transendothelial migration and cell adhesion were highly correlated with recurrence-free survival. The gene signature is highly predictive of recurrence in stage I NSCLC patients, which has important prognostic and therapeutic implications for the future management of these patients.

## Introduction

Lung cancer is still the leading cause of cancer-related death for both men and women in the United States, though therapeutic outcomes have gradually improved. In 2010, there were estimated 222,520 new cases of lung cancer diagnosed and only 15% of those will be alive after 5 years [1]. Non-small cell lung cancer (NSCLC) constitutes about 85% of all lung cancers, with small cell carcinoma making up the remaining 15%. About 25% to 30% of patients with NSCLC have stage I disease and receive surgical intervention alone. Despite undergoing curative surgery, more than 25% of patients with stage I NSCLC will die from recurrent disease within five years [2], [3]. Adjuvant cisplatin based chemotherapy in stage I–III NSCLC improves survival modestly following surgical resection [4], [5], [6]. Cancer and Leukemia Group B (CALGB) 9633, a phase III study that compared adjuvant therapy with carboplatin/paclitaxel versus surgery alone for completely resected stage IB NSCLC, showed a significant survival benefits to adjuvant therapy after 2.8 years of median follow-up [7] but not after 4.5 years of follow-up [8]. Reliable clinical or molecular prognostic factors, as well as guidelines for treatment of recurrent stage I NSCLC have not been well elucidated. Because of heterogeneity in recurrence rates among cancer patients with the same stage, it is critical to isolate a reliable molecular signature in tumors that could be used to identify those who are likely to develop recurrent disease and would thus benefit from adjuvant therapy. Moreover, identification of genes and molecular pathways critical for development of metastasis could lead to advances in therapeutics.

Advances in human genomics and proteomics have generated lists of candidate biomarkers with potential clinical values. Gene expression profiling has been used to characterize prognosis in lung cancer, mostly using overall survival (OS) rather than tumor recurrence as an end point [9], [10], [11], [12], [13], [14]. However, the identified survival-related genes lacked consistency among these studies, likely due to limited patient samples, disease heterogeneity, and/or technical factors such as differences in microarray platforms and specimen processing. Integrating microarray data from multiple studies to increase sample size holds promise for the development of more robust prognostic tests. We thus conducted a meta-analysis of seven data sets to search for differentially expressed genes related to overall survival time [15] and identified a 64-gene expression signature that is highly predictive of OS of stage I NSCLC patients. Our results indicate that gene expression signatures are useful in predicting survival of stage I lung cancer, and meta-analysis of microarray datasets increases statistical power for detecting survival-related differentially expressed genes.

In investigations of the effectiveness of adjuvant therapy, OS is considered as the gold standard end point. However, the disadvantage of OS is that it requires an extended follow-up. Recently several studies explored disease-free survival (DFS) as a possible alternative end point of OS. Some evidences had been offered for the use of DFS as a surrogate for OS in colorectal cancer, breast cancer and stomach cancer [16]. In these studies, the Pearson's correlation between 5-year OS and 3-year DFS was 0.97 and Spearman's rank correlation was 0.92; the Pearson's correlation between hazard ratios for OS and DFS was 0.85 and Spearman's rank correlation was 0.87.

In this study, we conducted a meta-analysis of microarray datasets from different institutions to develop and validate a novel gene-expression signature that can accurately predict tumor recurrence of stage I NSCLC patients. The identified signature has potential to refine the clinical practice in the management patients with resected NSCLC.

## Methods

### Data Collection

The Director's Challenge Consortium for the Molecular Classification of Lung Adencarcinoma (“Director's Challenge Consortium”) collected more than 300 lung adenocacinoma samples from four institutions (HLM, MICH, DFCI, and MSKCC) along with pertinent clinical data [17]. In our study we used a total of 142 patient samples with stage I lung adencarcinoma, which were not given adjunctive chemotherapy or radiotherapy, as training samples to identify a gene-expression signature for recurrence free survival. The data were downloaded from https://array.nci.nih.gov/caarray/project/details.action?project.experiment.publicIdentifier=jacob-00182.

Other four independent datasets (datasets 2–5) were used as testing samples for validation of the identified signature. Dataset 2 included 46 stage I lung adenocarcinomas. Dataset 3 included both adenocarcinomas and squamous cell carcinomas with 64% of 138 samples being stage I tumors. It is important to know whether our developed signature is applicable to other cancer subtype such as squamous cell carcinomas or not. Dataset 2 and 3 were downloaded from GEO database (GSE5843 and GSE8894). Dataset 4 was generated by Mayo Clinic and included 54 stage I NSCLC in never smokers, and most of them were adenocarcinomas. Dataset 5 was generated by our own group at Washington University which was used to identify our 64-gene signature for overall survival (the data was deposited in GEO database as GSE6253) [15]. All patients in these validation sets were not given adjuvant chemotherapy or radiotherapy.

PRISMA 2009 flow diagram regarding the dataset selection is showed in Figure S1. Details of the clinical information for the subjects in each dataset are described in Table 1. The endpoint was time to recurrence, defined as the time from surgical resection to the first evidence of tumor recurrence (local, regional or distant). Patients were censored from the recurrence analysis at the earliest of the following time points: death, development of second primary NSCLC, or last medical contact. The involved microarray platforms included Affymetrix Hu133A (dataset 1), Hu133plus2 (dataset 3), HG_U95Av2 array (dataset 5), 22 K Operon Human Genome Oligo Set v2.1 (http://www.operon.com) (dataset 2) and Illumina DASL assay (dataset 4).

**Table 1:** Clinical summary of patients in the analyzed datasets.

	Dataset 1	Dataset 2	Dataset 3	Dataset 4	Dataset 5
Total number of samples	142	46	138	54	36
Mean age (range)	65 (35–85)	63 (36–78)	61 (31–82)	69 (32–89)	66 (48–81)
Sex					
male	80	33	104	9	20
female	62	13	34	45	16
Mean follow-up (months)					
Total DFS	57	39	35	48	38
No recurred	69	63	54	55	51
Recurred	27	35	16	33	22
Stage					
IA	70	16	—	27	0
IB	72	30	—	27	36
Histological type					
ADC	142	46	62	49	14
SCC	0	0	76	1	18
Others	0	0	0	4	4

### Data Processing

Even though the training dataset is from one study, the samples were collected and profiled in four different institutions. Systematic differences in gene expression from these institutions may be remarkable, which would compromise the integrity of the data from different labs. The distance-weighted discrimination (DWD) method (https://genome.unc.edu/pubsup/dwd/index.html) was used to identify and adjust systematic biases that were present within this microarray dataset. The DWD method corrects for systematic biases across microarray batches by finding a separating hyperplane between the two batches and adjusting the data by projecting the different batches on the DWD plane, finding the batch mean, and then subtracting out the DWD plane multiplied by this mean [18].

### Statistical analysis

#### Identify differentially expressed genes related to recurrence

Multivariate Cox proportional hazards regression analyses (adjusted for age, gender, and cancer stage) with 10,000 bootstrap resampling were performed for each gene using all of the 142 samples in Dataset 1. The proportional hazards assumption for these variables was investigated by examining the scaled Schoenfeld residuals. The categorical variables gender and cancer stage displayed significant deviation from the proportional hazards assumption and were thus taken as strata in regression models. The genes were then ranked according to the bootstrap frequencies of P<0.01 for their gene expression in regression models. We then performed GO term enrichment analysis on these differentially expressed genes using the Database for Annotation, Visualization and Integrated Discovery (DAVID) bioinformatics resource (http://david.abcc.ncifcrf.gov/home.jsp). Similar statistical analyses were detailed in a previous study [15].

#### Define a gene-expression signature for recurrence

The following survival analyses were also based on all of the 142 samples in Dataset 1. Partial Cox regression method was performed to construct predictive components [19]. These components were then used in the Cox model for building predictive models for recurrence-free survival of cancer patients. The principle components were chosen in the model to maximize Somers' Dxy rank correlation. The risk scores were calculated by 
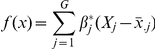
, where 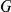 represents the number of genes; 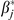 represents the estimated coefficient of the 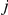
th gene; 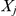 represent gene expression levels of the 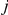
th gene in all the samples, 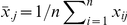 where 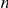 is sample size and 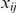 is the gene expression level of gene 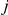 from sample 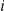. All the samples were classified into high and low risk groups according to the risk scores. Patients with risk scores less than zero potentially have long-term recurrence-free survivals and those larger than zero have short-term recurrence-free survival after surgical resection. To choose an appropriate subset of genes for signature, we carried out a forward selection procedure to optimize a gene-expression signature: 1) increase one gene each time based on the rank of genes that were identified in the above bootstrap analyses; 2) perform the partial Cox regression analysis and obtain the prediction accuracy using the chosen subset of genes; and 3) repeat steps 1 and 2 until the prediction accuracy is maximized. The prediction accuracy (discrimination ability) was assessed by Somers' Dxy rank correlation of estimated risk score and real survival time. Somers' Dxy is related to the C-index by Dxy = 2(C-0.5). C is the corresponding receiver operating characteristic (ROC) curve area, which is a graphical representation of the pairs of false-positive test results (specificity) and true-positive test results (sensitivity) for the realizations of a quantitative test.

To identify a gene signature robustly predicting time to recurrence, leave-one-out cross-validation (LOOCV) was used. Briefly, 142 iterations of the above forward selection procedure were performed so that each sample was left out once with a set of genes in relation to time to recurrence calculated at each iteration. The frequency of the genes occurring in the signatures were ranked to identify genes that consistently, and robustly, correlated with outcome. The genes that passed the set criterion (frequency >50%) were selected to comprise the final signature.

To evaluate the predictive performance of the proposed gene signature, we employed time-dependent ROC analysis for censored data and area under the curve (AUC) as our criteria to assess recurrence predictions. The time-dependent sensitivity and specificity functions are defined as: 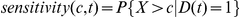 and 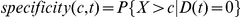. The corresponding ROC(t) curve for any time t is defined as the plot of {sensitivity(c, t)} versus {1 – specificity(c, t)}, with cutoff point c varying. X is the covariate and D(t) is the event indicator (here, recurrence) at time t. The area under the curve, AUC(t), is defined as the area under the ROC(t) curve. A nearest neighbor estimator for the bivariate distribution function is used for estimating these conditional probabilities accounting for possible censoring [20]. AUC can be used as an accuracy measure of the diagnostic marker; the larger the AUC, the better the prediction model. AUC = 0.5 indicates no predictive power, whereas AUC = 1 represents perfect predictive performance. Kaplan-Meier survival analyses were implemented after the samples were classified into two risk groups. Differences of the recurrent risk between the two risk groups were assessed using the Mantel-Haenszel log rank test. The larger area between the two risk groups and its associated smaller p value from the Mantel-Haenszel log rank test implicate a better classification model. Somers' Dxy rank correlation of estimated risk score and real survival time were also calculated.

#### Validate the signature in four independent microarray datasets

After the signature was defined, we evaluated it in four independent datasets (i.e., Datasets 2–5). The expression data of genes in the signature were used to estimate risk score for each samples in the independent datasets. Please note that the gene numbers used to estimate risk score were different because of the different microarray platforms used in the training dataset and testing datasets. The Partial Cox regression were redone for each dataset to get the estimated coefficient of each gene in order to calculate risk score for each sample. Somers' Dxy rank correlation of estimated risk score and real survival time were calculated and time-dependent ROC analysis were performed for each testing dataset.

#### Identify significant pathways related to recurrence

Partial Cox regression method was also performed for each KEGG pathway. The risk scores were calculated using the gene sets in each pathway. All the samples were classified into high and low risk groups according to the risk scores. Differences in recurrent risk between the two risk groups were assessed using the Mantel-Haenszel log rank test. P values less than 10^−4^ were use to define significant pathways.

All of the data analyses were implemented using the R statistical package (www.r-project.org).

## Results

### Differentially expressed genes associated with recurrence

To identify a gene expression signature of tumor recurrence, we analyzed a training set of 142 stage I lung adencarcinomas from the Director's Challenge Consortium, including 70 with stage IA (T1N0M0) disease and 72 with stage IB (T2N0M0). None of the142 patients in the analysis were given adjuvant chemotherapy or radiotherapy. Multivariate Cox proportional hazards regression analyses with bootstrap resampling approaches were performed for each gene to determine if it was significantly associated with cancer recurrence. We identified 104 probesets from 98 known genes with bootstrap frequencies greater than 80% for their gene expression in regression models (Table S1). Eighteen probesets were associated with good outcome (hazard ratio <1.0), that is, patients with higher expressions of these genes tend to have longer recurrence free survival. In contrast, the other 86 probesets were associated with bad outcome (hazard ratio >1.0), that is, increased expression of these genes result in shorter recurrence free survival of stage I patients. GO term enrichment analysis on these differentially expressed genes indicated one third of the genes we identified are potentially involved in known cancer-related pathways. Among them, *B4GALT1, CELSR1, CLDN4, CLDN9, COL2A1, ALCAM, ICAM4, MUC5AC and THBS1* are related to cell adhesion; *NLRP2*, *CGB, LUC7L3, ELMO2, EIF2AK2, IFI6, MUC5AC, NFKBIL1, PPT1, PACS2, RHOT1, THBS1* are related to apoptosis; and *CLEC11A, B4GALT1, BMP2, EIF2AK2, FABP3, FGFR2, ING1, ITCH, MUC5AC, NFKBIL1, THBS1, TCF3* are related to regulation of cell proliferation.

### Identification of a gene signature for recurrence in the training set

**Figure 1:**
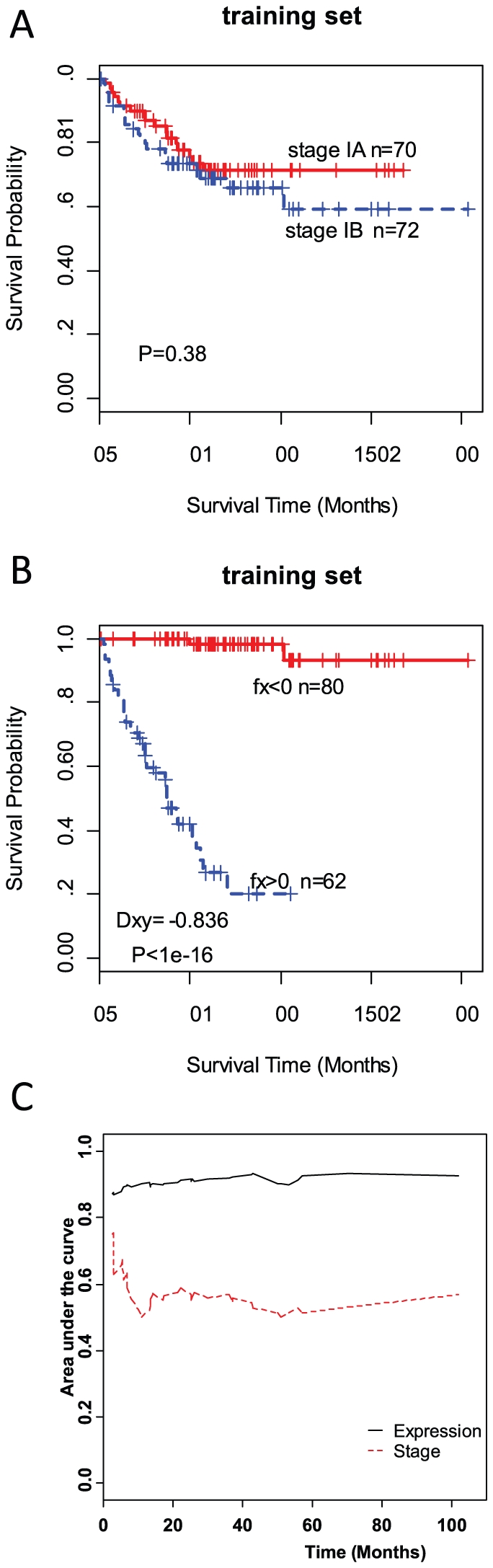
Survival analyses of the training set of 142 stage I denocarcinomas. (A) Kaplan-Meier survival curves for two groups of patients with stage IA or IB. (B) Kaplan-Meier survival curves for the two groups of patients defined by having positive (high risk) or negative (low risk) risk scores of recurrence-free survival. The risk scores were estimated with 15 principle components based on the model using 51 recurrence-free survival-related genes. (C) The area under the curve (AUC) of time-dependent ROC analysis for survival models based on stage information or 51-gene expression data respectively. Time is indicated in months on the x-axis, cumulative survival is indicated on the y-axis. Tick marks, patients whose data were censored at last follow-up.

Next, we attempted to identify a manageable, robust set of genes whose expression could be used to predict primary tumors likely to recur. We employed a partial Cox regression analysis with leave-one-out cross-validation in the training dataset of 142 stage I patients. In each cross-validation, we identified a gene signature that gives the highest prediction accuracy and recorded genes entered in the identified signature. We then counted the frequency of genes present in all of the cross-validation sets. Genes with a frequency >50% were selected to comprise the final signature (Table 2). Finally, risk scores were estimated for each of 142 samples in the training dataset using the expression data of these 51 genes. Based on the risk scores, we classified these patients into high and low risk groups and performed Kaplan-Meier survival analyses on these stratified samples. As shown in Fig. 1, recurrence-free survival was significantly different between the high and low-risk groups as defined by the risk scores using the expression data (P<1e-16). Kaplan-Meier survival curves could not distinguish poorer survival among stage IB from stage IA NSCLC (P = 0.38). To evaluate their predictive performance, we further calculated the time-dependent area under the ROC curves based on either stage information or the estimated risk scores of the patients (Fig. 1C). The expression-based stratified approach performs much better than the pathological staging method. Our approach achieves AUCs close to 90% while the Cox model with stage information results in very low AUCs<60%.

**Table 2:** Genes related to tumor recurrence of stage I NSCLC.

Genes	Function	HR	Genes	Function	HR
AU148154		0.5792	NM_018600		1.5353
B4GALT1	Cell adhesion	1.8344	OCA2	cell differentiation	1.4181
CGB	cell death	1.3312	PADI3	terminal differentiation of the epidermis	1.5470
CHST12		1.4697	RPRM	negative regulation of progression through cell cycle	1.4748
CLEC11A	positive regulation of cell proliferation	1.6334	SH3YL1		1.5522
COL2A1	negative regulation of apoptosis, Cell adhesion	1.5701	SLC27A2	PPAR signaling pathway	1.4456
CYP2A6	nicotine metabolism	1.2751	SLC35F5		1.4836
DENND1A	synaptic vesicle endocytosis	1.4545	SNAPC2	transcription from RNA polymerase II promoter	1.5725
DIO1		1.5142	SPTBN2	cell death	1.6520
DOCK6		1.6545	STRN3		1.3969
EPHB6	Loss of expression in metastatic melanoma	1.4146	SUSD4		1.4464
FZD9	G-protein coupled receptor protein signaling pathway	1.2810	TCF3	transcription factor activity	1.5250
GLE1	export mRNA from nucleus to cytoplasm	1.4920	TET3	tet oncogene family member 3	1.6322
GTF3C2	transcription factor	1.6350	THBS1	Cell adhesion, blood vessel development	1.3397
INF2	Rho GTPase binding	1.4114	TRIM34		1.4886
KDM4B	transcriptional target of hypoxia-inducible factor	1.7967	TRIM46		1.4355
SIK3	protein phosphorylation	0.5875	TRIP11	transcription from RNA polymerase II promoter	1.4917
GREB1L		1.4917	CELSR1	Cell adhesion	1.5144
KLK5	epidermis development	1.4736	UBE2D4	ubiquitination	1.4669
KRT81	keratin filament	1.3167	UBXN4	response to unfolded protein	1.4742
LENEP	cell differentiation	1.5902	VKORC1	oxidoreductase activity	1.5498
MYOG	cell differentiation	1.6048	ZBTB7B	cell differentiation	1.5783
NFKBIL1	member of the I-kappa-B family	1.5875	ZNF365		1.5436
NLRP2	cell death	1.4080	MUC5AC	induction of apoptosis, Cell adhesion	1.4135
NM_004876			FGFR2	cell growth	1.5516
FEZ2	cell projection organization and biogenesis	1.6395			

### Validation of the recurrence signature in independent test sets

To determine if the 51-gene signature could predict patients likely to develop tumor recurrence in independent samples, we applied it to four independent datasets (Table 1). Specifically, a risk score for each patient was calculated based on the expression levels of the 51-gene signature; poor outcome was defined as risk score >0 and good outcome was defined as risk score <0. Cox proportional hazards modeling was used to classify patients in each of the testing datasets. The predictive accuracy of the recurrence signature was determined by AUC of time-dependent ROC analysis and Somers' Dxy rank correlation between estimated risk score and real survival time.

**Figure 2:**
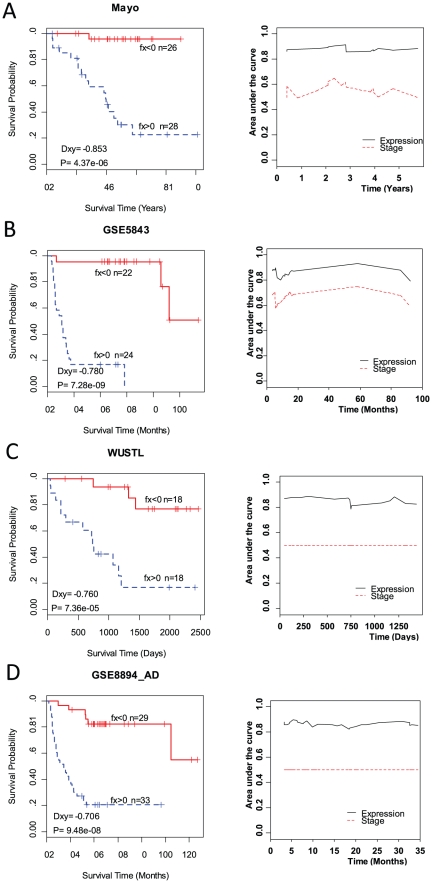
Validation of the 51-gene signature in four independent datasets. Kaplan-Meier survival analysis was performed in low (*full red line*) and high (*dashed blue line*) risk patient groups defined by the 51-gene classifier. AUC for survival models based on stage (*dashed red line*) or 51-gene classifier (*full black line*) was also compared. The testing dataset GSE8894 do not have available stage information and all patients in the WUSTL dataset are stage IB. So the time dependent ROC using stage information in these two datasets could not be calculated; all set at 0.5 instead. Tick marks, patients whose data were censored at last follow-up.

Mayo Clinic dataset included 54 never smokers with stage I NSCLC, and most of which were adenocarcinomas. The risk scores estimated by expression of 46 genes presented on Illumina DASL assay have high correlation with the real survival time (Dxy = −0.853). AUC from time-dependent ROC analysis is about 88% using the risk scores and 57% using stage information. Predicted poor-outcome patients had a significantly worse recurrence-free survival (log-rank *P* = 4.37e−6) (Fig. 2A). In the testing dataset GSE5843 with 46 stage I adenocarcinoma, the gene signature has an overall accuracy of 86% and the predicted high risk scores are significantly associated with shorter observed time to recurrence (log-rank P = 7e−9; Fig. 2B). In contrast, the accuracy of predicting recurrence using stage information alone is 66%.

Only 32 of 51 genes in the recurrence signature are available on the early-generation Affymetrix U95A microarray used in the WUSTL testing dataset. Despite incomplete representation, the signature we identified still has a good performance with the AUCs around 85% in predicting recurrence. Kaplan-Meier analysis confirmed that the predicted high-risk group have a significantly shorter time to recurrence than the low-risk group (P = 7.36e−5) (Fig. 2C).

GSE8894 is the largest testing dataset, including 62 adenocarcinomas and 76 squamous cell carcinomas. We evaluated the performance of our signatures in predicting recurrence-free survival in adenocarcinomas and squamous cell carcinomas separately. Cox model with risk scores estimated by expression data give a good predictive performance (Dxy = −0.706) with the AUCs of more than 85% for adenocarcinoma (Fig. 2D). In squamous cell carcinomas, we obtained a little less predictive AUCs, but the predicted high-risk group still had a significantly shorter time to recurrence (Dxy = −0.678 and P = 3.48e−7, Fig. S2).

### Significant pathways related to recurrence

Pathway-based survival analyses identified 97 significant prognostic KEGG pathways related to recurrence (p<10^−5^, Table S2). Table 3 listed the top 30 important pathways, including multiple important cancer-related pathways such as cell adhesion molecules, the Jak-STAT signaling pathway, p53 signaling pathway, MAPK signaling pathway, Wnt signaling pathway, mTOR signaling pathway and ErbB signaling pathway. The differentially expressed genes associated with recurrence identified by our survival analysis were also enriched in biological process of cell adhesion.

**Table 3:** Top 30 significant prognostic KEGG pathways related to recurrence.

KEGGpathway	Pathway annotation	Genenumber	P value
hsa04670	Leukocyte transendothelial migration	116	1.01E-13
hsa04141	Protein processing in endoplasmic reticulum	166	3.99E-13
hsa04514	Cell adhesion molecules (CAMs)	113	7.23E-12
hsa00230	Purine metabolism	161	1.44E-11
hsa03013	RNA transport	151	2.77E-11
hsa04630	Jak-STAT signaling pathway	155	3.14E-11
hsa03040	Spliceosome	127	3.82E-11
hsa04660	T cell receptor signaling pathway	108	6.73E-11
hsa04722	Neurotrophin signaling pathway	127	1.11E-10
hsa04144	Endocytosis	195	1.24E-10
hsa04380	Osteoclast differentiation	128	1.29E-10
hsa04730	Long-term depression	69	1.68E-10
hsa04115	p53 signaling pathway	68	2.96E-10
hsa00190	Oxidative phosphorylation	132	3.62E-10
hsa04010	MAPK signaling pathway	267	4.65E-10
hsa04910	Insulin signaling pathway	138	7.16E-10
hsa04930	Type II diabetes mellitus	48	8.82E-10
hsa04060	Cytokine-cytokine receptor interaction	264	1.10E-09
hsa04530	Tight junction	132	2.48E-09
hsa04666	Fc gamma R-mediated phagocytosis	92	3.75E-09
hsa04310	Wnt signaling pathway	150	4.61E-09
hsa04020	Calcium signaling pathway	177	4.84E-09
hsa04150	mTOR signaling pathway	52	5.24E-09
hsa03008	Ribosome biogenesis in eukaryotes	77	5.49E-09
hsa03015	mRNA surveillance pathway	83	5.62E-09
hsa03010	Ribosome	91	5.78E-09
hsa04914	Progesterone-mediated oocyte maturation	86	7.37E-09
hsa05322	Systemic lupus erythematosus	122	9.24E-09
hsa04120	Ubiquitin mediated proteolysis	135	9.66E-09
hsa04012	ErbB signaling pathway	87	1.08E-08

## Discussion

A major limitation of current clinical prognostic indicators is their inability to predict which patients with early-stage disease will develop disease recurrence. We previously described a 64-gene signature of overall survival in stage I NSCLC capable of predicting outcome in independent samples [15]. In this study, we sought to determine if a comparable signature existed in stage I adenocarcinomas to predict recurrence-free survival in lung cancer. Using microarray datasets of stage I lung cancer from the Director's Challenge Consortium, we further developed a new gene-expression signature predictive of recurrence of stage I NSCLC patients. We used samples from four institutions in the Director's Challenge Consortium as the training dataset to identify a gene-expression signature for lung cancer recurrence. To reduce disease heterogeneity and confounding effects from treatments, we used a total of 142 stage I lung adencarcinomas patients without adjunctive chemo- or radiation-therapy as the training samples (Table 1). To integrate the gene expression data from the four institutions, we applied DWD method to remove systematic differences that were present within this dataset. Subsequently, we identified 104 genes whose expression was correlated with recurrence-free survival. As expected, the gene ontology composition of these genes has biological relevance to disease recurrence, such as cell adhesion, apoptosis, and cell proliferation.

Using a partial Cox regression model-based forward selection procedure, we identified a 51-gene signature from 104 differentially expressed genes. The identified signature is highly predictive of tumor recurrence in patients with stage I lung adenocarcinomas. One of the potential issues in developing a predictive signature is model overfitting to the training dataset. This may result in a signature that reflects the characteristics of the training samples and cannot accurately predict outcome in independent samples. To avoid model overfitting, we further used leave-one-out cross-validation procedure to generate the gene signature of recurrence in the training dataset. Consequently, it is also critical to validate the prediction signature in independent datasets. We therefore applied our signature in four independent datasets to evaluate its prediction performance. In general, our signature is highly predictive of which patients with stage I lung adenocarcinomas will develop recurrence disease and it achieves more than 85% in AUC across different independent datasets. The test set GSE8894 included both adenocarcinomas and squamous cell carcinomas; 36% of samples were advanced stage patients. A recent study showed that lung cancer recurrence depends on histological subtype in the stage IA non-small cell lung cancer, with higher rates occurring among patients with non-squamous carcinomas [21]. Interestingly, the 51-gene signature was also highly predictive of recurrence free survival of squamous cell carcinomas in the dataset GSE8894 although it was initially derived from stage I adenocarcinoma.

The identified differentially expressed genes in the present study may provide new insights into therapeutic targets and treatment of recurrence disease in stage I lung tumors. Among them, FBXW7 targets mTOR for degradation and cooperates with PTEN in tumor suppression [22]. The low FBXW7 expression group showed a significantly poorer prognosis than in the high expression group in patients with colorectal cancer [23]. Its lower expression were also associated with decreased recurrence-free survival in stage I lung adenocarcinomas (Table S1). Another interesting candidate is FGFR2, which is one of transmembrane tyrosine kinase receptors involved in signaling via interaction with the fibroblast growth factor (FGF) family. The fibroblast growth factor (FGF) family, which includes important regulatory factors of cell growth and differentiation, has been found to be involved in embryonic development, angiogenesis and tumorigenesis. It has been suggested that FGFR2 plays an important role in the tumorigenesis of gastric cancer. We found the increased expression in FGFR2 is associated with poor outcome of stage I lung cancer patients. A newly developed small-molecule-acting FGFR inhibitor, Ki23057, can compete with ATP for the binding site in the kinase [24]. It will be interesting to see if such an inhibitor can improve the outcome of patients who are predicted to be at a high-risk of recurrence with the gene-expression signature. In addition, we also identified three splicing factors SFRS2IP, SFRS14 and SFRS18 associated with disease outcome. All three splicing factors are members of the arginine/serine-rich family and worthy of further study.

Our pathway-based survival analyses found that leukocyte transendothelial migration, protein processing in endoplasmic reticulum and cell adhesion molecules (CAMs) are the top three KEGG pathways highly correlated with recurrence-free survival (Fig. S3). It's not a surprise that these three pathways are all significantly related to recurrence. Leukocytes cross the endothelium lining the vasculature initiated by chemokine- and adhesion molecule-induced intracellular signaling that controls adhesion, spreading, and motility. At the same time, adherent leukocytes trigger the endothelium, manipulating the barrier to promote their transmigration into the underlying tissues [25]. CAMs are gatekeepers for leukocyte transendothelial migration. Endothelial cell intercellular CAMs expression is negatively correlated with metastatic potential in lung cancer [26]. L1 cell adhesion molecule (L1CAM) has potential prognostic value in pulmonary neuroendocrine tumors. Patients with high L1 expression have a higher risk for recurrence compared with those with low L1 expression [27]. The endoplasmic reticulum (ER) is an essential organelle involved in many cellular functions including protein folding and secretion. The ER plays a vital role in cellular protein quality control by extracting and degrading proteins that are not correctly folded or assembled into native complexes, i.e. ER-associated degradation (ERAD) to ensure that only properly folded and assembled proteins are transported to their final destinations. The ER is also a major organelle for oxygen and nutrient sensing as cells adapt to their microenvironment. The unfolded protein response (UPR) is a cellular stress response related to the ER. It is activated in response to an accumulation of unfolded or misfolded proteins in the lumen of the ER. In this scenario, the UPR has two primary aims: initially to restore normal function of the cell by halting protein translation and activate the signaling pathways that lead to increased production of molecular chaperones involved in protein folding. If these objectives are not achieved within a certain time lapse or the disruption is prolonged, the UPR leads to apoptosis [28]. Cell adhesion molecules (CAMs) pathway is much more important than cell cycle and apoptosis in prognosis of recurrence according to our results (Table 3). Determination of CAMs expression as biomarker in future clinical trials may be widely realized for cancer therapy.

In summary, we identified a 51-gene expression signature highly predictive of tumor recurrence in stage I NSCLC and validated it in four independent data sets. This gene expression signature has the potential of the identification of high-risk individuals who would perhaps benefit most from adjuvant treatment in early-stage lung cancers. If patients predicted to be at high risk of recurrent disease by genomic signatures are shown in clinical trials to be those who benefit most from adjuvant treatment, the clinical pay-off for genomic tumor analyses will have been realized.

## Supporting Information

Figure S1 **PRISMA flow diagram.** PRISMA 2009 flow diagram regarding the data selection. The databases used for the search of microarray data were GEO, ArrayExpress and caARRAY. In the research, we used the keywords: “lung”, “cancer”, “gene” and “survival”.
(EPS)

Figure S2 **Validation of the 51-gene signature in the subset of squamous cell carcinomas in GSE8894.** Comparison of survival estimates in low (*full red line*) and high (*dashed blue line*) risk patient groups defined by the 51-gene classifier is shown in the left panel. AUC for survival models based on 51-gene classifier (*full black line*) is shown in the right panel. The stage information is not available in GSE8894, and the AUC was set at 0.5. Tick marks, patients whose data were censored at last follow-up.
(EPS)

Figure S3 **KEGG pathway-based survival analyses in the training set of 142 stage I adenocarcinomas.** Kaplan-Meier survival analysis was performed in low and high risk patient groups stratified by the risk scores that were estimated from the expression of genes in each pathway. (A) Leukocyte transendothelial migration, (B) Protein processing in endoplasmic reticulum, and (C) Cell adhesion molecules. Time is indicated in months on the x-axis, cumulative survival is indicated on the y-axis. Tick marks, patients whose data were censored at last follow-up.
(EPS)

Table S1 **Differentially expressed genes related to recurrence.**
(DOCX)

Table S2 **Significant KEGG pathways related to recurrence.**
(DOCX)
