# Supplementary material for: Gene-Expression Signature Predicts Postoperative Recurrence in Stage I Non-Small Cell Lung Cancer Patients
Source: PLoS One. 2012 Jan 23;7(1):e30880. doi: 10.1371/journal.pone.0030880 (PMC3264655; doi:10.1371/journal.pone.0030880)
Supplement: Table S1 — Differentially expressed genes related to recurrence. (DOCX) [file pone.0030880.s004.docx]

**Table S1 Differentially expressed genes related to recurrence**

| probe | Gene Symbol | Pvalue | HR | Lower .95 | Upper .95 |
| --- | --- | --- | --- | --- | --- |
| 215616_s_at | KDM4B | 2.26E-05 | 1.7967 | 1.3701 | 2.3559 |
| 208942_s_at | SEC62 | 4.06E-05 | 0.4500 | 0.3074 | 0.6589 |
| 216627_s_at | B4GALT1 | 0.000132 | 1.8344 | 1.3439 | 2.5038 |
| 221044_s_at | TRIM34 | 0.000198 | 1.4886 | 1.2073 | 1.8356 |
| 206448_at | ZNF365 | 0.000208 | 1.5436 | 1.2272 | 1.9416 |
| 215000_s_at | FEZ2 | 0.000287 | 1.6395 | 1.2551 | 2.1415 |
| 210620_s_at | GTF3C2 | 0.000376 | 1.6350 | 1.2470 | 2.1437 |
| 217877_s_at | GPBP1L1 | 0.000447 | 1.7256 | 1.2725 | 2.3401 |
| 221115_s_at | LENEP | 0.000634 | 1.5902 | 1.2187 | 2.0750 |
| 213850_s_at | SFRS2IP | 0.000784 | 0.5237 | 0.3590 | 0.7639 |
| 212442_s_at | LASS6 | 0.000785 | 1.6998 | 1.2472 | 2.3167 |
| 222242_s_at | KLK5 | 0.000786 | 1.4736 | 1.1751 | 1.8478 |
| 212115_at | HN1L | 0.000802 | 1.5381 | 1.1958 | 1.9784 |
| 218751_s_at | FBXW7 | 0.000814 | 0.4169 | 0.2498 | 0.6958 |
| 218672_at | SCNM1 | 0.000833 | 1.4891 | 1.1789 | 1.8808 |
| 216920_s_at | TARP | 0.000862 | 0.3290 | 0.1711 | 0.6327 |
| 205769_at | SLC27A2 | 0.000929 | 1.4456 | 1.1623 | 1.7980 |
| 41660_at | CELSR1 | 0.000942 | 1.5144 | 1.1842 | 1.9367 |
| 204718_at | EPHB6 | 0.001049 | 1.4146 | 1.1496 | 1.7407 |
| 209744_x_at | ITCH | 0.001052 | 1.6405 | 1.2200 | 2.2058 |
| 204019_s_at | SH3YL1 | 0.001213 | 1.5522 | 1.1893 | 2.0258 |
| 204415_at | IFI6 | 0.001213 | 1.5374 | 1.1848 | 1.9949 |
| 221563_at | DUSP10 | 0.001238 | 1.5711 | 1.1944 | 2.0666 |
| 212778_at | PACS2 | 0.001403 | 1.3684 | 1.1288 | 1.6587 |
| 209376_x_at | SFRS2IP | 0.001406 | 0.5324 | 0.3616 | 0.7839 |
| 212517_at | ATRN | 0.001463 | 0.4986 | 0.3248 | 0.7655 |
| 209797_at | CNPY2 | 0.001467 | 0.4752 | 0.3005 | 0.7515 |
| 205155_s_at | SPTBN2 | 0.001483 | 1.6520 | 1.2121 | 2.2514 |
| 213622_at | COL9A2 | 0.001532 | 1.3690 | 1.1273 | 1.6625 |
| 34406_at | PACS2 | 0.001536 | 1.3130 | 1.1094 | 1.5539 |
| 208835_s_at | LUC7L3 | 0.001585 | 0.4828 | 0.3072 | 0.7586 |
| 221709_s_at | ZNF839 | 0.001656 | 1.6082 | 1.1962 | 2.1622 |
| 212446_s_at | LASS6 | 0.001657 | 1.5315 | 1.1743 | 1.9974 |
| 207493_x_at | SSX2 | 0.001691 | 1.3230 | 1.1109 | 1.5756 |
| 215243_s_at | GJB3 | 0.001712 | 1.4293 | 1.1434 | 1.7868 |
| 203153_at | IFIT1 | 0.001712 | 1.4114 | 1.1380 | 1.7506 |
| 206921_at | GLE1 | 0.001722 | 1.4920 | 1.1617 | 1.9160 |
| 209778_at | TRIP11 | 0.001768 | 1.4917 | 1.1609 | 1.9168 |
| 214754_at | TET3 | 0.001808 | 1.6322 | 1.1998 | 2.2204 |
| 210017_at | MALT1 | 0.001937 | 0.5484 | 0.3751 | 0.8018 |
| 217404_s_at | COL2A1 | 0.001944 | 1.5701 | 1.1803 | 2.0886 |
| 204211_x_at | EIF2AK2 | 0.001975 | 1.5796 | 1.1824 | 2.1101 |
| 205131_x_at | CLEC11A | 0.002000 | 1.6334 | 1.1966 | 2.2296 |
| 207194_s_at | ICAM4 | 0.002134 | 1.3999 | 1.1294 | 1.7352 |
| 214635_at | CLDN9 | 0.002145 | 1.5983 | 1.1847 | 2.1562 |
| 217949_s_at | VKORC1 | 0.002153 | 1.5498 | 1.1715 | 2.0503 |
| 220779_at | PADI3 | 0.002235 | 1.5470 | 1.1695 | 2.0464 |
| 205157_s_at | KRT17 | 0.002269 | 1.3386 | 1.1100 | 1.6143 |
| 65521_at | UBE2D4 | 0.002270 | 1.4669 | 1.1470 | 1.8762 |
| 204914_s_at | SOX11 | 0.002300 | 1.5018 | 1.1563 | 1.9505 |
| 213361_at | TDRD7 | 0.002306 | 1.4633 | 1.1455 | 1.8692 |
| 217502_at | IFIT2 | 0.002325 | 1.4162 | 1.1320 | 1.7718 |
| 221794_at | DOCK6 | 0.002357 | 1.6545 | 1.1960 | 2.2887 |
| 205387_s_at | CGB | 0.002466 | 1.3312 | 1.1061 | 1.6021 |
| 208952_s_at | LARP4B | 0.002589 | 1.4513 | 1.1390 | 1.8493 |
| 209973_at | NFKBIL1 | 0.002634 | 1.5875 | 1.1747 | 2.1454 |
| 213026_at | ATG12 | 0.002647 | 0.5440 | 0.3658 | 0.8091 |
| 222148_s_at | RHOT1 | 0.002668 | 1.5308 | 1.1595 | 2.0212 |
| 219461_at | PAK6 | 0.002809 | 1.4643 | 1.1402 | 1.8806 |
| 218927_s_at | CHST12 | 0.002864 | 1.4697 | 1.1411 | 1.8929 |
| 218144_s_at | INF2 | 0.002907 | 1.4114 | 1.1250 | 1.7707 |
| 207282_s_at | MYOG | 0.002916 | 1.6048 | 1.1753 | 2.1912 |
| 206498_at | OCA2 | 0.002942 | 1.4181 | 1.1265 | 1.7852 |
| 201952_at | ALCAM | 0.003017 | 1.5275 | 1.1545 | 2.0210 |
| 200975_at | PPT1 | 0.003041 | 1.3952 | 1.1194 | 1.7389 |
| 215505_s_at | STRN3 | 0.003176 | 1.3969 | 1.1187 | 1.7441 |
| 55692_at | ELMO2 | 0.003282 | 0.5890 | 0.4139 | 0.8383 |
| 218387_s_at | PGLS | 0.003292 | 1.5932 | 1.1678 | 2.1735 |
| 204104_at | SNAPC2 | 0.003319 | 1.5725 | 1.1625 | 2.1272 |
| 213711_at | KRT81 | 0.003349 | 1.3167 | 1.0956 | 1.5825 |
| 211295_x_at | CYP2A6 | 0.003379 | 1.2751 | 1.0838 | 1.5001 |
| 215392_at | AU148154 | 0.003510 | 0.5792 | 0.4014 | 0.8357 |
| 220909_at | TRIM46 | 0.003520 | 1.4355 | 1.1260 | 1.8299 |
| 213505_s_at | SFRS14 | 0.003598 | 0.5826 | 0.4050 | 0.8382 |
| 206457_s_at | DIO1 | 0.003731 | 1.5142 | 1.1439 | 2.0042 |
| 221690_s_at | NLRP2 | 0.003769 | 1.4080 | 1.1170 | 1.7748 |
| 212006_at | UBXN4 | 0.003878 | 1.4742 | 1.1328 | 1.9184 |
| 205853_at | ZBTB7B | 0.003945 | 1.5783 | 1.1573 | 2.1525 |
| 211398_at | FGFR2 | 0.003995 | 1.5516 | 1.1505 | 2.0925 |
| 201107_s_at | THBS1 | 0.004126 | 1.3397 | 1.0970 | 1.6360 |
| 206277_at | P2RY2 | 0.004136 | 1.5315 | 1.1444 | 2.0495 |
| 220340_at | GREB1L | 0.004177 | 1.4917 | 1.1346 | 1.9611 |
| 204156_at | SIK3 | 0.004184 | 0.5875 | 0.4082 | 0.8454 |
| 212599_at | AUTS2 | 0.004305 | 1.4925 | 1.1338 | 1.9648 |
| 220879_at | --- | 0.004305 | 1.5353 | 1.1438 | 2.0606 |
| 201166_s_at | PUM1 | 0.004305 | 1.4539 | 1.1245 | 1.8798 |
| 219763_at | DENND1A | 0.004358 | 1.4545 | 1.1242 | 1.8817 |
| 207639_at | FZD9 | 0.004428 | 1.2810 | 1.0801 | 1.5193 |
| 219370_at | RPRM | 0.004500 | 1.4748 | 1.1280 | 1.9281 |
| 213771_at | IRF2BP1 | 0.004541 | 1.3682 | 1.1019 | 1.6990 |
| 214303_x_at | MUC5AC | 0.004569 | 1.4135 | 1.1128 | 1.7955 |
| 208415_x_at | ING1 | 0.004661 | 0.5600 | 0.3748 | 0.8368 |
| 219389_at | SUSD4 | 0.004680 | 1.4464 | 1.1200 | 1.8680 |
| 205738_s_at | FABP3 | 0.004702 | 1.3949 | 1.1074 | 1.7570 |
| 205289_at | BMP2 | 0.004712 | 0.4119 | 0.2226 | 0.7620 |
| 220123_at | SLC35F5 | 0.004714 | 1.4836 | 1.1285 | 1.9505 |
| 204915_s_at | SOX11 | 0.004773 | 1.4138 | 1.1116 | 1.7982 |
| 205290_s_at | BMP2 | 0.004795 | 0.4061 | 0.2171 | 0.7596 |
| 204913_s_at | SOX11 | 0.004819 | 1.4842 | 1.1279 | 1.9531 |
| 206862_at | ZNF254 | 0.004903 | 1.3846 | 1.1038 | 1.7370 |
| 212051_at | WIPF2 | 0.004903 | 1.4452 | 1.1182 | 1.8678 |
| 201428_at | CLDN4 | 0.004905 | 1.5241 | 1.1363 | 2.0442 |
| 212177_at | SFRS18 | 0.004928 | 0.5404 | 0.3518 | 0.8299 |
| 216647_at | TCF3 | 0.005000 | 1.5250 | 1.1358 | 2.0476 |
