# Supplementary material for: Gene-Expression Signature Predicts Postoperative Recurrence in Stage I Non-Small Cell Lung Cancer Patients
Source: PLoS One. 2012 Jan 23;7(1):e30880. doi: 10.1371/journal.pone.0030880 (PMC3264655; doi:10.1371/journal.pone.0030880)
Supplement: Table S2 — Significant KEGG pathways related to recurrence. (DOCX) [file pone.0030880.s005.docx]

**Table S2 Significant KEGG pathways related to recurrence**

| KEGG  pathway | Pathway annotation | Gene  number | P value |
| --- | --- | --- | --- |
| hsa04670 | Leukocyte transendothelial migration | 116 | 1.01E-13 |
| hsa04141 | Protein processing in endoplasmic reticulum | 166 | 3.99E-13 |
| hsa04514 | Cell adhesion molecules (CAMs) | 113 | 7.23E-12 |
| hsa00230 | Purine metabolism | 161 | 1.44E-11 |
| hsa03013 | RNA transport | 151 | 2.77E-11 |
| hsa04630 | Jak-STAT signaling pathway | 155 | 3.14E-11 |
| hsa03040 | Spliceosome | 127 | 3.82E-11 |
| hsa04660 | T cell receptor signaling pathway | 108 | 6.73E-11 |
| hsa04722 | Neurotrophin signaling pathway | 127 | 1.11E-10 |
| hsa04144 | Endocytosis | 195 | 1.24E-10 |
| hsa04380 | Osteoclast differentiation | 128 | 1.29E-10 |
| hsa05200 | Pathways in cancer | 325 | 1.55E-10 |
| hsa04730 | Long-term depression | 69 | 1.68E-10 |
| hsa04115 | p53 signaling pathway | 68 | 2.96E-10 |
| hsa00190 | Oxidative phosphorylation | 132 | 3.62E-10 |
| hsa04010 | MAPK signaling pathway | 267 | 4.65E-10 |
| hsa04910 | Insulin signaling pathway | 138 | 7.16E-10 |
| hsa04930 | Type II diabetes mellitus | 48 | 8.82E-10 |
| hsa05222 | Small cell lung cancer | 85 | 9.91E-10 |
| hsa04060 | Cytokine-cytokine receptor interaction | 264 | 1.10E-09 |
| hsa04530 | Tight junction | 132 | 2.48E-09 |
| hsa04666 | Fc gamma R-mediated phagocytosis | 92 | 3.75E-09 |
| hsa04310 | Wnt signaling pathway | 150 | 4.61E-09 |
| hsa04020 | Calcium signaling pathway | 177 | 4.84E-09 |
| hsa04150 | mTOR signaling pathway | 52 | 5.24E-09 |
| hsa03008 | Ribosome biogenesis in eukaryotes | 77 | 5.49E-09 |
| hsa03015 | mRNA surveillance pathway | 83 | 5.62E-09 |
| hsa03010 | Ribosome | 91 | 5.78E-09 |
| hsa04914 | Progesterone-mediated oocyte maturation | 86 | 7.37E-09 |
| hsa04120 | Ubiquitin mediated proteolysis | 135 | 9.66E-09 |
| hsa04012 | ErbB signaling pathway | 87 | 1.08E-08 |
| hsa04621 | NOD-like receptor signaling pathway | 58 | 1.34E-08 |
| hsa04070 | Phosphatidylinositol signaling system | 78 | 1.34E-08 |
| hsa04360 | Axon guidance | 129 | 1.40E-08 |
| hsa04062 | Chemokine signaling pathway | 189 | 1.77E-08 |
| hsa05223 | Non-small cell lung cancer | 54 | 2.12E-08 |
| hsa04145 | Phagosome | 134 | 2.32E-08 |
| hsa04650 | Natural killer cell mediated cytotoxicity | 131 | 2.48E-08 |
| hsa04510 | Focal adhesion | 200 | 2.63E-08 |
| hsa04912 | GnRH signaling pathway | 100 | 2.91E-08 |
| hsa04662 | B cell receptor signaling pathway | 75 | 3.32E-08 |
| hsa04620 | Toll-like receptor signaling pathway | 102 | 3.32E-08 |
| hsa04622 | RIG-I-like receptor signaling pathway | 71 | 5.08E-08 |
| hsa04142 | Lysosome | 121 | 7.18E-08 |
| hsa04623 | Cytosolic DNA-sensing pathway | 56 | 1.13E-07 |
| hsa00983 | Drug metabolism - other enzymes | 52 | 1.15E-07 |
| hsa04110 | Cell cycle | 124 | 1.40E-07 |
| hsa04540 | Gap junction | 90 | 1.60E-07 |
| hsa04740 | Olfactory transduction | 388 | 1.67E-07 |
| hsa04971 | Gastric acid secretion | 74 | 1.78E-07 |
| hsa04916 | Melanogenesis | 101 | 1.79E-07 |
| hsa04350 | TGF-beta signaling pathway | 84 | 1.86E-07 |
| hsa00561 | Glycerolipid metabolism | 50 | 2.13E-07 |
| hsa04664 | Fc epsilon RI signaling pathway | 78 | 2.70E-07 |
| hsa04640 | Hematopoietic cell lineage | 83 | 2.76E-07 |
| hsa01100 | Metabolic pathways | 1128 | 3.25E-07 |
| hsa00240 | Pyrimidine metabolism | 98 | 4.52E-07 |
| hsa00980 | Metabolism of xenobiotics by cytochrome P450 | 71 | 4.62E-07 |
| hsa05215 | Prostate cancer | 88 | 5.49E-07 |
| hsa05120 | Epithelial cell signaling in Helicobacter pylori infection | 68 | 7.89E-07 |
| hsa04612 | Antigen processing and presentation | 56 | 8.27E-07 |
| hsa04972 | Pancreatic secretion | 100 | 9.08E-07 |
| hsa05220 | Chronic myeloid leukemia | 73 | 9.24E-07 |
| hsa05210 | Colorectal cancer | 62 | 9.52E-07 |
| hsa00020 | Citrate cycle (TCA cycle) | 30 | 9.80E-07 |
| hsa00562 | Inositol phosphate metabolism | 57 | 1.08E-06 |
| hsa03050 | Proteasome | 44 | 1.13E-06 |
| hsa05100 | Bacterial invasion of epithelial cells | 70 | 1.20E-06 |
| hsa00640 | Propanoate metabolism | 32 | 1.57E-06 |
| hsa05014 | Amyotrophic lateral sclerosis (ALS) | 54 | 1.66E-06 |
| hsa00051 | Fructose and mannose metabolism | 36 | 1.69E-06 |
| hsa05150 | Staphylococcus aureus infection | 41 | 2.05E-06 |
| hsa05217 | Basal cell carcinoma | 55 | 2.17E-06 |
| hsa04964 | Proximal tubule bicarbonate reclamation | 23 | 2.33E-06 |
| hsa04114 | Oocyte meiosis | 112 | 2.47E-06 |
| hsa05221 | Acute myeloid leukemia | 57 | 2.69E-06 |
| hsa00380 | Tryptophan metabolism | 42 | 2.71E-06 |
| hsa04370 | VEGF signaling pathway | 75 | 2.73E-06 |
| hsa05332 | Graft-versus-host disease | 21 | 2.73E-06 |
| hsa04720 | Long-term potentiation | 70 | 2.79E-06 |
| hsa00350 | Tyrosine metabolism | 41 | 2.98E-06 |
| hsa00280 | Valine, leucine and isoleucine degradation | 44 | 4.03E-06 |
| hsa00310 | Lysine degradation | 44 | 4.20E-06 |
| hsa05212 | Pancreatic cancer | 70 | 4.25E-06 |
| hsa04520 | Adherens junction | 73 | 4.89E-06 |
| hsa04810 | Regulation of actin cytoskeleton | 213 | 4.93E-06 |
| hsa00565 | Ether lipid metabolism | 35 | 5.13E-06 |
| hsa04146 | Peroxisome | 79 | 5.19E-06 |
| hsa03320 | PPAR signaling pathway | 70 | 5.22E-06 |
| hsa00520 | Amino sugar and nucleotide sugar metabolism | 48 | 6.07E-06 |
| hsa00564 | Glycerophospholipid metabolism | 81 | 6.64E-06 |
| hsa04920 | Adipocytokine signaling pathway | 68 | 7.12E-06 |
| hsa05214 | Glioma | 65 | 7.26E-06 |
| hsa00270 | Cysteine and methionine metabolism | 36 | 8.29E-06 |
| hsa04962 | Vasopressin-regulated water reabsorption | 44 | 9.08E-06 |
| hsa04610 | Complement and coagulation cascades | 69 | 9.14E-06 |
| hsa03420 | Nucleotide excision repair | 46 | 9.79E-06 |
